# Contribution of Fe_3_O_4_ nanoparticles to the fouling of ultrafiltration with coagulation pre-treatment

**DOI:** 10.1038/srep13067

**Published:** 2015-08-13

**Authors:** Wenzheng Yu, Lei Xu, Nigel Graham, Jiuhui Qu

**Affiliations:** 1 Key Laboratory of Drinking Water Science and Technology, Research Center for Eco-Environmental Sciences, Chinese Academy of Sciences, Beijing 100085, China; 2 Department of Civil and Environmental Engineering, Imperial College London, South Kensington Campus, London SW7 2AZ, UK; 3 Centre for Water Resources Research (CWRR), School of Civil, Structural and Environmental Engineering, University College Dublin, Newstead Building, Belfield, Dublin 4, Ireland

## Abstract

A coagulation (FeCl_3_)-ultrafiltration process was used to treat two different raw waters with/without the presence of Fe_3_O_4_ nanoparticle contaminants. The existence of Fe_3_O_4_ nanoparticles in the raw water was found to increase both irreversible and reversible membrane fouling. The trans-membrane pressure (TMP) increase was similar in the early stages of the membrane runs for both raw waters, while it increased rapidly after about 15 days in the raw water with Fe_3_O_4_ nanoparticles, suggesting the involvement of biological effects. Enhanced microbial activity with the presence of Fe_3_O_4_ nanoparticles was evident from the measured concentrations of extracellular polymeric substances (EPS) and deoxyribonucleic acid (DNA), and fluorescence intensities. It is speculated that Fe_3_O_4_ nanoparticles accumulated in the cake layer and increased bacterial growth. Associated with the bacterial growth is the production of EPS which enhances the bonding with, and between, the coagulant flocs; EPS together with smaller sizes of the nano-scale primary particles of the Fe_3_O_4_-CUF cake layer, led to the formation of a lower porosity, more resilient cake layer and membrane pore blockage.

Ultrafiltration (UF) membrane systems are being applied increasingly in the treatment of ground waters and surface waters owing to their ability to produce high quality drinking water economically, particularly for the removal of bacteria and viruses. However, the presence of natural organic matter (NOM) in such waters, typically comprising a complex mixture of humic and fulvic acids, proteins, and carbohydrates, is believed to be the major membrane foulant^1^,^2^. Reasonably good correlations have been shown between the presence of these organic substances and the rate of irreversible fouling for long-term UF operations, especially with respect to the biopolymer content, such as protein-like and polysaccharide-like substances^3^,^4^. Some researchers have concluded that after organic matter, ferric oxide and silica are the next most common foulants, followed by alumina and calcium sulphate^5^.

A trend of increasing use of commercial nanoparticles is evident in recent years, and there is growing concern about associated environmental problems as they are synthesized and used on a large scale, leading to the contamination of natural water bodies, such as by the release of water-dispersive modified Fe_3_O_4_ nanoparticles^6^. For example, some nanoparticles incorporated into clothing articles can be released into water, and subsequently influence the operation of wastewater treatment plants (WWTPs)^7^. Of additional concern is that the nanoparticles can sorb or incorporate toxic metals and carry toxins downstream^8^. Therefore, removing these nanoparticles from contaminated water is of great importance, and membrane filtration, as a particle separation process, is in principle an effective method. However, it is possible that nanoparticles may exacerbate membrane fouling as the sizes of nanoparticles are close to that of ultrafiltration membrane pores.

In order to control membrane fouling and remove nanoparticles, many methods have been used to prevent the organic fouling materials and nanoparticles reaching the membrane, such as ion exchange (IX)^9^ and coating iron oxide^10^. “In-line” chemical coagulation or coagulation–hydraulic flocculation, has been shown to be an effective way of not only improving general water quality, but of controlling membrane fouling^11^,^12^,^13^. The addition of polyaluminum chloride had a positive impact in reducing hydraulically irreversible fouling by these constituents^3^. However, the coagulation process produces a cake layer, and many experimental studies and practical operations have indicated that cake layer formation is the main cause of membrane fouling^14^,^15^,^16^. Therefore, the characteristics of the cake layer should be explored, and particularly the presence of extracellular polymeric substances (EPS) and bacteria.

There are many species of nanoparticles having antibacterial activity on bacteria, such as copper oxide/copper iodide nanoparticles^17^,^18^, TiO_2_^19^ and ZnO^20^. If the nano ferric oxide can kill bacteria during the coagulation and ultrafiltration processes, it may improve the coagulation-ultrafiltration performance. However, iron oxide particles have been found to enhance microorganism growth^21^. Iron-reducing bacteria with a wide variety of morphologies were associated with nano-aggregates, indicating that cell surface Fe(III) accumulation may be a general mechanism by which iron-reducing bacteria can grow^22^, using a wide range of organic compounds as electron donors^23^. Spherical α-Fe_2_O_3_ nanoparticles (~50 nm) is indicative of a Morin-like transition and there is a translation between Fe_2_O_3_ and Fe_3_O_4_ in some conditions^24^. Also Fe(III) solid phases are the products of Fe(II) oxidation by Fe(II)-oxidizing bacteria, but the Fe(III) phases reported to occur during the growth experiments were poorly crystalline in nature^25^.

Therefore, it can be concluded that the existence of Fe(II)-Fe(III) nanoparticles in coagulated surface water, polluted by Fe_3_O_4_ nanoparticles, may influence/enhance microorganism growth and thereby affect membrane performance. Up to now, there has been no published research that has established whether the existence of Fe_3_O_4_ nanoparticles mitigates or aggravates membrane fouling. Thus, in this paper we describe the results of mini-pilot-scale tests that were undertaken to explore the contribution of Fe_3_O_4_ nanoparticles in polluted raw water to the fouling of a UF membrane with Fe(III) pre-coagulation; the study involved the quantification of changes in the nature of the organic constituents (e.g. EPS, NOM), the properties of the cake layer, and other relevant parameters.

## Methods and Materials

### Synthetic raw water and coagulant

A synthetic, polluted raw water with/without Fe_3_O_4_ nanoparticles (2 mg/L) was used for the tests. The raw water was based on tap water supplemented with domestic settled sewage and humic acid in order to simulate a surface water supply slightly polluted by sewage discharge, and containing soluble microbial products. Thus, domestic settled sewage was added to the local (Beijing, China) tap water with a volumetric ratio of 1:50, together with 5 mg/L (around 2 mg/L TOC) of Suwannee River humic acid (Standard II, International Humic Substances Society, St. Paul, MN, USA)^26^, which was similar in nature to Suwannee River NOM^27^. Prior to mixing with domestic sewage and humic acid solution, the tap water was left for one night to ensure the complete decay of residual chlorine. The characteristics of the synthetic raw water are listed in Table 1. Ferric chloride was used as the coagulant in this study, and stock FeCl_3_ solutions were prepared at a concentration of 0.05 M in deionized (DI) water.

### Two pretreatments before UF processes

**Figure 1:**
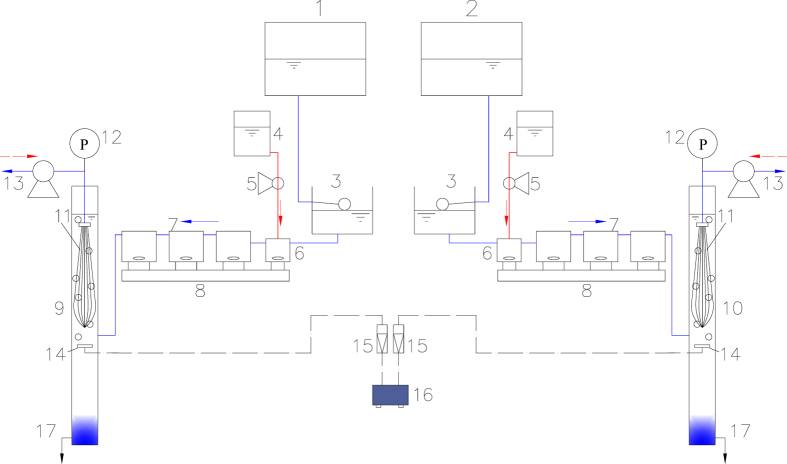
Schematic diagram of the experimental set-up (1—raw water tank (without Fe_3_O_4_ nanoparticles) 2—raw water tank (with Fe_3_O_4_ nanoparticles); 3—constant level water tank; 4—FeCl_3_ tank; 5—mini-peristaltic pump; 6—mixing system; 7– flocculation system; 8—magnetic stirrer with showing stirring speed; 9—CUF tank; 10—FeNP-CUF tank; 11—membrane module; 12—pressure gauge; 13—suction/backwash peristaltic pump; 14—air blower; 15– air flowmeter; 16—air diffuser; 17—sludge discharge.

The experimental study was based on two parallel UF systems incorporating conventional coagulation and flocculation by FeCl_3_ before ultrafiltration, treating the synthetic raw water without (CUF), and with, Fe_3_O_4_ nanoparticles (FeNP-CUF). For the latter, the raw water was mixed with 2 mg/L Fe_3_O_4_ nanoparticles (around 20 nm in size), which were prepared by the same method of our colleagues^28^. A schematic illustration of the CUF and FeNP-CUF arrangements is shown in Fig. 1. The two synthetic raw waters were fed into constant-level tanks to maintain the water head for the membrane tanks. A constant dose of FeCl_3_ (0.1 mM) was continuously added into the rapid mixing tanks. The rapid mix speed was 200 rpm (G = 184 s^−1^) in the mixing tank with a hydraulic retention time (HRT) of 1 min, which then reduced to 50 rpm (23 s^−1^) in the three subsequent flocculation tanks, each having a HRT of 5 min. For both systems, the water after flocculation was passed directly to the membrane tank. Each tank contained a submerged polyvinylidene fluoride (PVDF) hollow-fiber UF membrane module (Tianjin Motimo Membrane Technology Co., Ltd, China) with a nominal pore size of 0.03 μm and a surface area of 0.025 m^2^ (inner diameter = 0.7 mm and outer diameter = 1.1 mm). The permeate through the submerged membrane module was continuously withdrawn using a peristaltic pump at a constant flux of 20 L.m^−2^.h^−1^, operated in a cycle of 30 min filtration and 1 min backwash (40 L.m^−2^.h^−1^). Air was supplied to each reactor at 100 L/h (air: water = 200:1) only at every backwash. The trans-membrane pressure (TMP) was continuously monitored using pressure gauges. The HRT of the membrane tanks was maintained at 0.5 h and accumulated sludge was released every day. The duration of the ultrafiltration tests was 30 days, and the membrane module was taken out and washed by sponge at day 25. The membrane filtration tests were operated throughout at a relatively stable temperature (around 27 ^o^C).

Further information concerning the pre-coagulation experiments before membrane filtration and the size distribution of particles in the membrane tanks can be found in a previous paper^29^.

### Samples of cake layer and sludge

At the end of the UF test period, the fouled membrane modules were taken out from the membrane tanks. The external foulants on the membrane surface were carefully scraped off with a plastic sheet (Deli, China) and simultaneously flushed with their influent waters from the membrane tanks. Then the cake layer and sludge were centrifuged at 3000 rpm for 10 min.

Some of the collected wet samples (0.5 g) after centrifugation were fully mixed in phosphate buffer saline (PBS) solution, and the mixed liquor was then heated to 80 ^o^C for 1 h. The heated liquor was cooled at room temperature and centrifuged at 10,000 g for 20 min, and the supernatant was filtered by 0.45 μm membrane and used as the bound EPS fraction for chemical analyses, which was according to Wang *et al.*^30^ and Yu *et al.*^31^. The EPS was extracted in duplicates for each sample.

After the membrane surface was wiped with a sponge, 0.01 mol/L NaOH was used for the extraction of internal foulants and the fibers were soaked for 24 h at 20 ^o^C in the alkaline solution according to the method described by Kimura *et al.*^32^. The organic matter that was extracted as described above was subject to the following chemical analyses.

The EPS concentration and deoxyribonucleic acid (DNA) analyses were described in a previous paper^33^, as well as the use of three-dimensional excitation-emission matrix (EEM) fluorescence spectroscopy (FP-6500, Jasco, Japan) for some of the samples.

### Other analytical methods

The UV absorbance at 254 nm, UV_254_, of 0.45 μm filtered solutions was determined by an ultraviolet/visible spectrophotometer (U-3010, Hitachi High Technologies Co., Japan). Dissolved organic carbon (DOC) was determined with a total organic carbon (TOC) analyzer (TOC-V_CPH_, Shimadzu, Japan). Residual Fe and total P after 0.45 μm membrane was measured by inductivity coupled plasma optical emission spectrometer (ICP-OES, 710, Agilent Technologies, USA). Residual turbidity (Hach 2100, USA) and floc size (Mastersizer 2000, Malvern, U.K.) measurements were conducted for samples in the two membrane tanks. The concentration of NH_4_^+^-N was determined by the colorimetric method using a spectrometer, and the concentrations of NO_3_^−^ were measured by Ion Chromatography (ICS-2000, Dionex, USA).

Subsamples of cake layer and new sludge after freeze drying were mounted in 1-mm-thick glass sample holders for X-ray diffraction (XRD, X-Pert PRO MPD, Philips, Netherlands) analysis, and diffraction patterns were recorded by unresolved Cu-Κα radiation at 40 kV and 40 mA. The magnetic properties of the flocs and cake layer were studied using a vibrating sample magnetometer (LakeShore 7307, USA) at room temperature by cycling the field from −10000 to 10000 kOe.

The fouled membrane fibers were cut from the two membrane modules, and the foulant layer attached on the membrane surface was retained on the membrane surface. The new and fouled membrane samples were then platinum-coated by a sputter and observed under scanning electron microscopy (SEM; JSM7401F, JEDL, Japan).

## Results

### Floc size and structure

**Figure 2:**
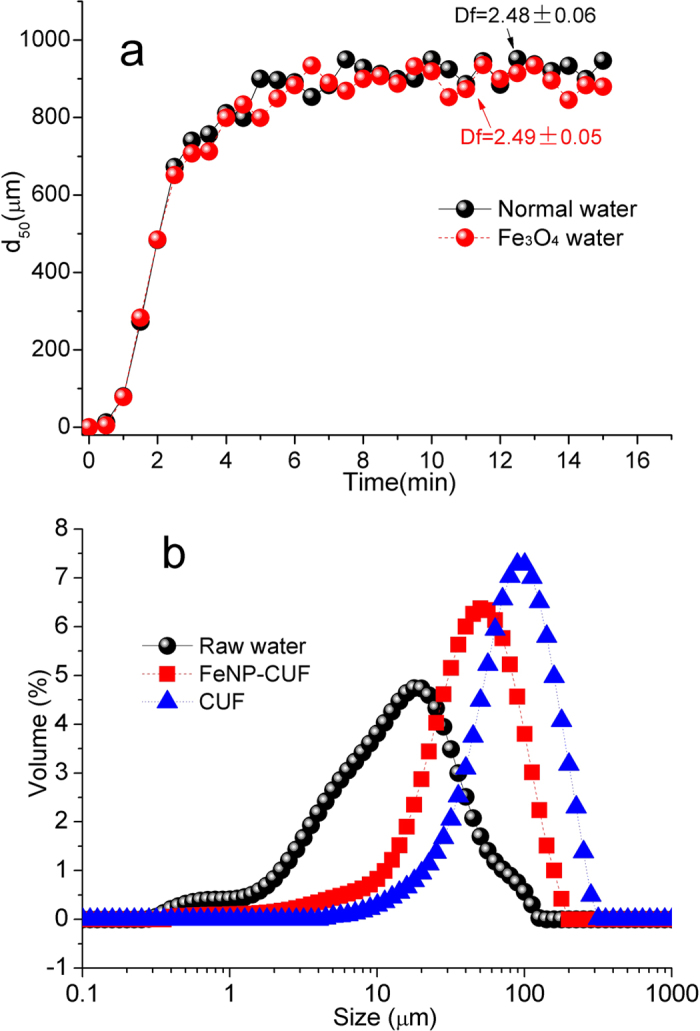
Coagulation performances (by FeCl_3_) for treating two raw waters in terms of the median floc size (d_50_) (**a**), and the size distribution of flocs in the two membrane systems (**b**).

The structure of the cake layer on the membrane surface can influence the external membrane fouling, and it is determined by the floc size and structure. From observations of the coagulation process and kinetics (e.g. Fig. 2a), it was evident that the floc formation, in terms of size and structure, were similar for the two raw waters (with and without Fe_3_O_4_ nanoparticles). This result meant that the presence of the Fe_3_O_4_ nanoparticles did not change the properties of the flocs significantly, which was consistent our previous work^34^. However, the greater density of Fe_3_O_4_ nanoparticles in the flocs improves their sedimentation/separation within the membrane tank, and it results in a greater quantity of small particles present in the FeNP-CUF tank (Fig. 2b). As a consequence, it seems likely that fewer flocs would attach onto the surface of the FeNP-CUF membrane, compared to the CUF membrane. However, the cake layer of FeNP-CUF membrane still contained Fe_3_O_4_ nanoparticles, which accumulated during the membrane operation, and it was able to influence the presence and activity of bacteria, as discussed subsequently.

### EEM and concentration of organic matter

**Figure 3:**
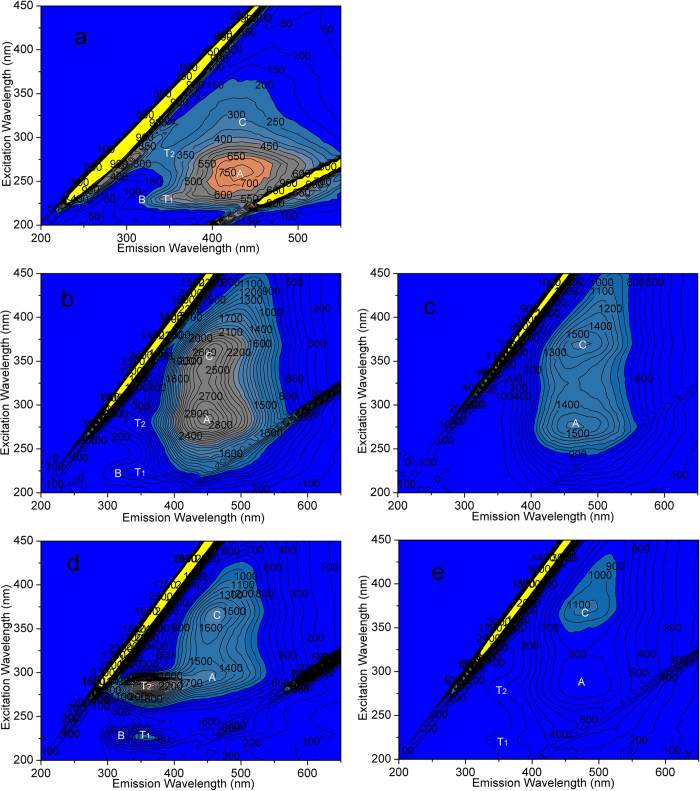
EEM spectra of organic matter from raw water (**a**) and the top sludge in the FeNP-CUF tank (**b**) and the CUF tank (**c**), and from the cake layers in the FeNP-CUF tank (**d**) and the CUF tank (**e**).

In order to confirm the existence of EPS in the cake layer and sludge, especially in the FeNP-CUF system, the results of the EEM analysis and EPS concentration were considered. The EEM fluorescence spectra of foulants extracted from the cake layer and upper layers of the sludge in the FeNP-CUF and CUF systems are shown in Fig. 3, and compared with the spectra of the raw water. Five fluorescence peaks were evident in the raw water as can be seen in Fig. 3a, and among these, peaks A and C are related to humic-like substances derived from the breakdown of plant material. In addition, protein-like fluorophores including tryptophan-like (Peak T) and tyrosine-like (Peak B) materials are usually detected at enhanced levels in water impacted by domestic sewage^35^.

The characteristics of the EEM fluorescence spectra of the top sludges from the two systems differed significantly from the raw water. For the sludge in the FeNP-CUF tank, there were also five peaks evident, similar to the raw water, but the strength of peak C was much greater which may be related to the retention of organic matter or the action of bacteria. For the CUF tank, in comparison, there were only two significant peaks, which suggested that there was a lower level of bacterial activity, as there was little indication of protein-like peaks (peak T_1_ and T_2_). The results indicated that the protein-like substances in FeNP-CUF sludge were enhanced by the existence of Fe_3_O_4_ nanoparticles, most probably produced by the bacteria or adsorbed by the cake layer from the raw water.

By comparing the EEM fluorescence spectra of the cake layers of the two membrane systems (Fig. 3d,e), all the five main peaks were similarly located with a difference of no more than 5 nm along the two axes, but the fluorescence intensities of the EEM peaks in FeNP-CUF system were much greater than those in the CUF system, which showed that greater quantities of protein-like materials were present in the cake layer of the former system. The fluorescence intensities of all the main EEM peaks associated with the cake layer and sludge from the FeNP-CUF were much greater than those from the CUF (especially for peak T_1_ and T_2_). Comparing the EEM peaks of material from the cake layer with that from the sludge in both membrane systems, the relative intensity of peaks A and C was less for the cake layers. It is expected that humic-like substances are readily separated within the coagulant floc, and hence accumulate within the settled sludge, and are probably utilized by bacteria in the cake layer. In contrast, the relative intensities of peaks T and B, relating to protein-like substances produced by bacteria, were greater in the cake layer materials, especially in the FeNP-CUF system. The observed, greater membrane fouling in the FeNP-CUF system is consistent with the findings of Hong *et al.*^36^ and Drews *et al.*^37^, who reported that proteins could induce severe membrane fouling as one of the major components of the membrane foulants.

**Figure 4:**
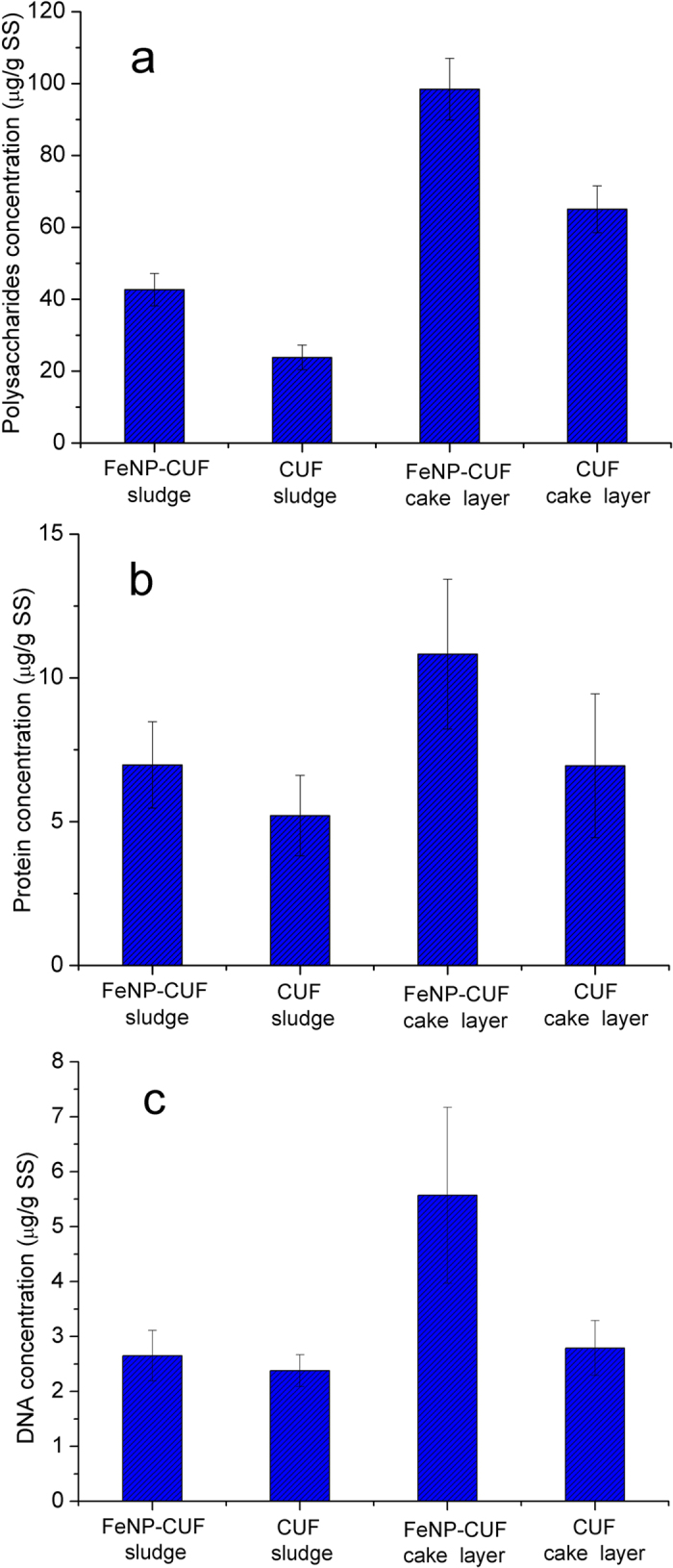
The concentration of polysaccharide (**a**), protein (**b**) and DNA (**c**) in the cake layers and sludges of the membrane units.

The EEM spectra of EPS can only measure relative concentration, and therefore the absolute EPS concentrations in the cake layers and sludges were also investigated (Fig. 4), to further explain their effect on membrane fouling. Bound EPS are composed of a variety of organic substances^38^, of which the main substances are polysaccharide and protein. Also, the concentration of bacteria was measured here, expressed in terms of DNA, as the bound EPS were produced by the bacteria.

Comparing the EPS content extracted from the sludges and cake layers in the two systems, it is clear that a greater EPS concentration was found in the cake layers for both systems (Fig. 4). Yu *et al.* obtained similar results in their experiments involving wastewater treatment^31^. For the CUF system the amount of bound polysaccharide in the cake layer (55 μg/gSS) was substantially greater than that in the sludge (24 μg/gSS), while in the Fe_3_O_4_-CUF system, the corresponding amounts were 98 μg/gSS and 42 μg/gSS. These results indicated that the concentrations of polysaccharides in the FeNP-CUF system were much higher than those in the CUF system. The variation of protein extracted from the cake layers and sludges in the two membrane systems was very similar to that of polysaccharide. Comparing the EEM patterns of the natural organic matter extracted from the cake layer and sludge with that of the EPS concentration, there was good agreement in terms of the presence of protein-like substances. According to the results shown in Figs 3 and 4a,b, a large increase of the EPS (polysaccharides and proteins) concentrations during the operation period would probably induce the large increase of TMP in FeNP-CUF.

The results of the DNA analysis showed that while the FeNP-CUF sludge only had a slightly greater DNA concentration than the CUF sludge, the quantity of DNA in the FeNP-CUF cake layer was approximately double that in the CUF cake layer (Fig. 4c). It is speculated that the presence of Fe_3_O_4_ nanoparticles enhanced the growth of bacteria using organic matter as nutrition. The presence of Fe_3_O_4_ nanoparticles, accumulating in the Fe(OH)_3_ cake layer, may provide a readily available electron acceptor for bacterial respiration, thereby leading to increased bacterial growth. An association between the presence of Fe_3_O_4_ nanoparticles and increased levels of bacteria has been reported by many researchers (e.g. Hanzlik *et al.*^39^; Jing *et al.*^40^). Banfield *et al.*^41^ found that microorganisms catalyze iron oxidation (Fe_3_O_4_) in acidic and near-neutral solutions, leading to accumulations of iron oxyhydroxides. Some bacteria can separate magnetic nanoparticles and apportion them equally between daughter cells, containing crystals of magnetite (Fe_3_O_4_) and/or greigete (Fe_3_S_4_), and other magnetic minerals^42^. Therefore, it is probable that in our tests Fe_3_O_4_ nanoparticles contributed to enhanced bacterial growth and activity, and thus greater EPS concentrations, which together led to the observed increase in membrane fouling.

### Magnetization and XRD pattern of cake layer and sludge

**Figure 5:**
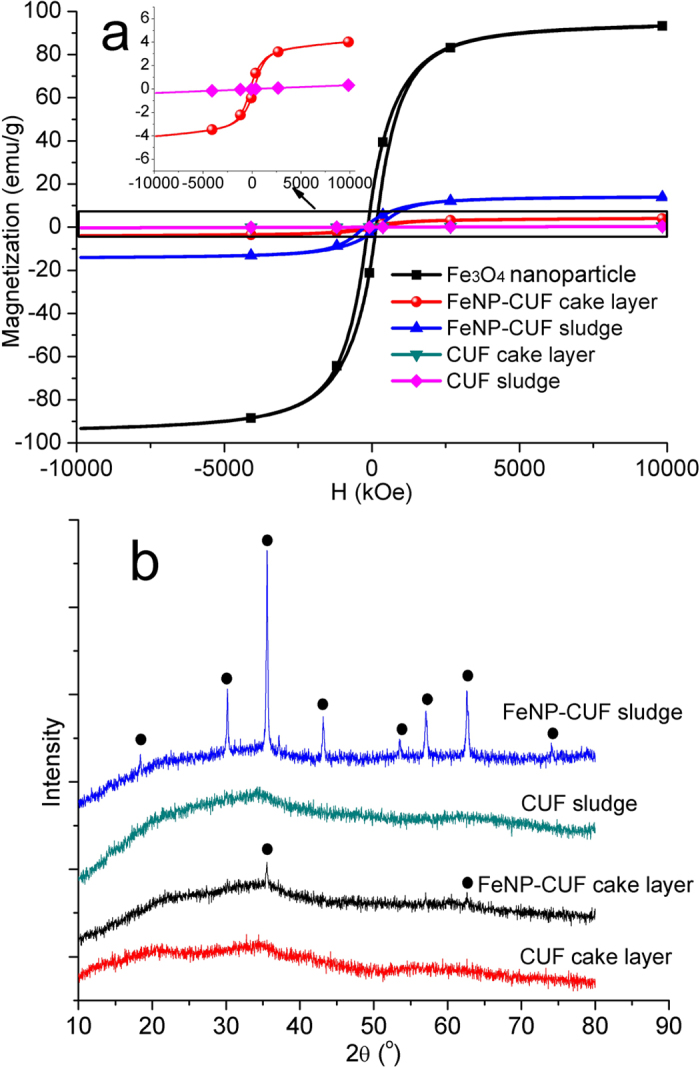
Magnetic field-dependent behaviour (**a**) and XRD pattern (**b**) of cake layers and sludges in the membrane tanks.

It is believed that the enhanced bacterial activity in the FeNP-CUF system changed the nature of the Fe_3_O_4_ nanoparticles to other materials during the membrane operation. Figure 5a shows the magnetic field-dependent behaviour of pure Fe_3_O_4_ nanoparticles, and the respective cake layers and sludges in the FeNP-CUF and CUF systems after freeze-drying. The sludge and cake layer in the CUF tank demonstrated no magnetic behaviour, while those in the FeNP-CUF tank had a ferromagnetic behaviour with a relatively wide magnetic hysteresis loop. The saturation magnetization, Ms, could be obtained by extrapolating the graph of M vs. 1/H to 1/H → 0 (for H > 2000 kOe). Although the magnetic strength of Fe_3_O_4_ sludge was much lower than that of raw Fe_3_O_4_ nanoparticles, the magnetic behaviour of Fe_3_O_4_ sludge (Ms value ∼ 13.8 emu/g) clearly showed that Fe_3_O_4_ nanoparticles were in the floc. The much lower magnetic behaviour of the FeNP-CUF cake layer (∼2.02 emu/g) indicated that these flocs were not only adsorbed onto the surface of cake layer, but also transformed into other materials which contained little magnetization, as a consequence of bacterial activity.

The XRD patterns of the cake layers and sludges were used to further confirm the results (Fig. 4b). There were no peaks for the CUF sludge and cake layer, which meant that the cake layer and sludge materials were amorphous in structure. In contrast, for the FeNP-CUF sludge and cake layer, there were clear peaks evident from the XRD patterns, and especially for the FeNP-CUF sludge sample. Comparing these peaks with the XRD pattern of pure Fe_3_O_4_ nanoparticles (Figure S1), there are clear similarities which indicate that the cake layer and sludge contained Fe_3_O_4_ nanoparticles. The decrease of peak density in the cake layer compared to the sludge in the FeNP-CUF system suggests the transformation of Fe_3_O_4_ nanoparticles to other materials. These results further confirmed that increased membrane fouling was caused by the growth of bacteria associated with the presence of mixed-valence Fe species coexisting in the precipitated aggregates^22^.

### SEM images

**Figure 6:**
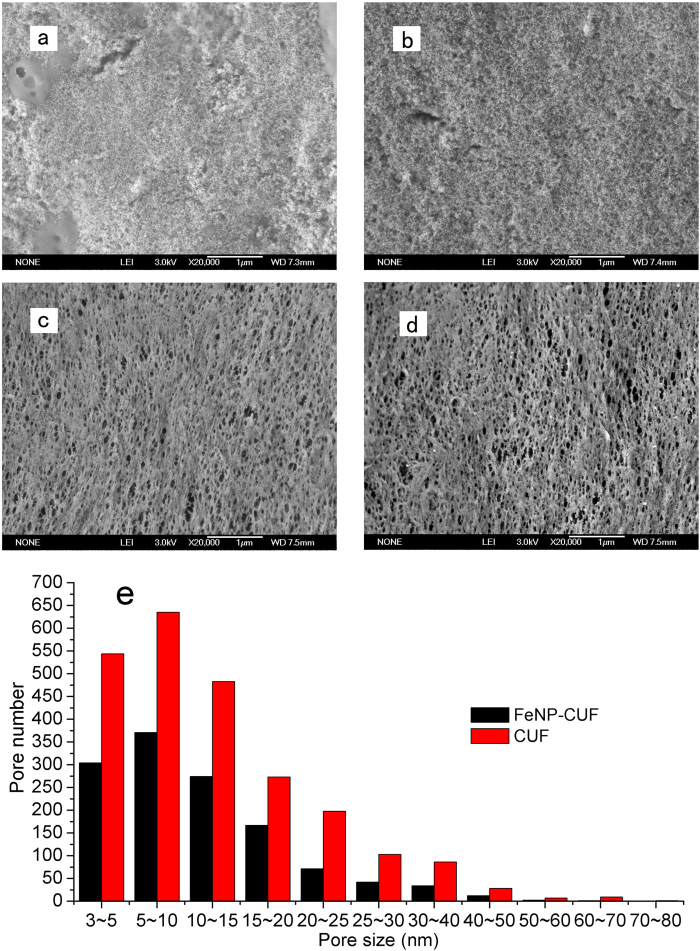
SEM images and status of pores for the membrane surfaces with different pretreatments: FeNP-CUF (**a**) and CUF (**b**) without washing, FeNP-CUF (**c**) and CUF (**d**) with washing; distribution of ‘open’ pores for washed membranes (**e**).

**Figure 7:**
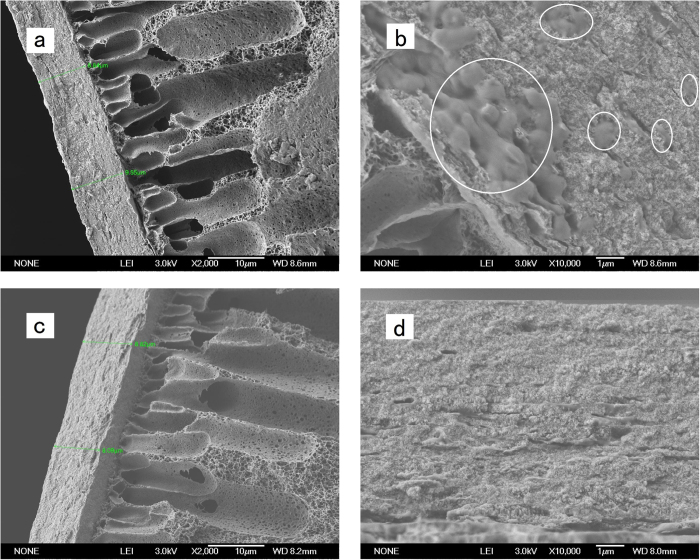
SEM images of the cross-section of cake layers on the membrane surface with different pretreatments: FeNP-CUF (**a**,**b**) and CUF (**c**,**d**).

In addition to the analytical methods described above, SEM images were also used to characterize the membrane after operation. Figures 6 and 7 present images of fouled and washed UF membranes, and their cross-sections. For the clean membrane there were a great number of large pores within the membrane surface and the pore distribution appeared relatively uniform, which has been reported in our previous research^29^. In contrast, used membranes displayed a thick deposit layer over the surface of the membranes, and the differences in the appearance of the cake layers deposited on the two membrane surfaces were apparent (Fig. 6). Some evidence of Fe_3_O_4_ nanoparticles on the surface of the FeNP-CUF cake layer was believed to be indicated by the SEM image, together with some EPS or other organic matter. Both systems contained thousands of colloidal deposits, most of which were considered to be precipitated nano-scale primary particles. For the CUF system membrane, the size of the nanoparticle precipitates seemed larger, and the cake layer appeared relatively more porous, compared to the FeNP-CUF system. It is speculated that the organic matter adsorbed on the nanoparticles (FeNP-CUF system) are used as nutrition for the microorganisms, thereby resulting in an apparently smaller size of nanoparticles.

**Figure 8:**
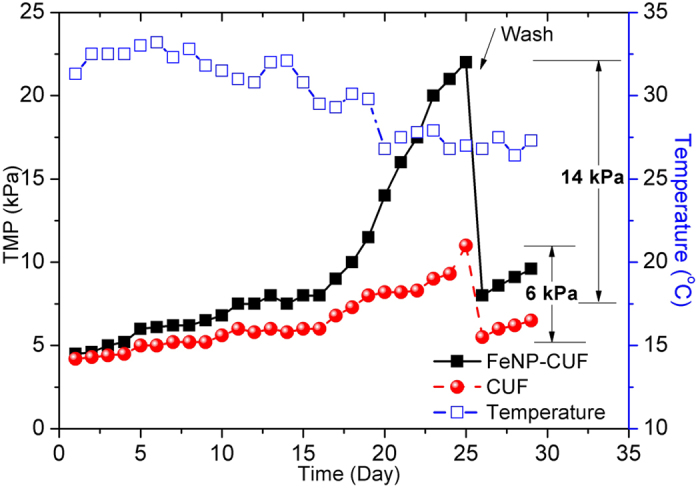
Temporal variation of TMP for coagulated raw water with and without Fe_3_O_4_ nanoparticles (FeNP-CUF and CUF, respectively) (20 L.m^−2^.h^−1^).

After washing of the membranes to remove the cake layer, images of the membrane surface were taken to explore the internal membrane fouling. As shown in Fig. 6c,d, the number of pores on the two fouled membrane surface seemed to be significantly different. It can be seen that large pores scarcely existed on the FeNP-CUF membrane surface and the number of pores had decreased to a large extent, whereas many large pores were clearly visible on the CUF membrane surface, and the statistical number of pores appeared to be much more than the FeNP-CUF membrane (Fig. 6e). The results of these microscopic observations demonstrated that internal fouling induced by the deposition or blockage of the pores of the FeNP-CUF membrane was much more serious than the CUF membrane, and this was consistent with the results of the internal fouling resistances as indicated by the TMP of both systems (after high pressure water wash at day 25) (Fig. 8).

Comparing the thickness of the cake layer formed in the two systems, it is evident that this was nearly the same in the two membrane systems (Fig. 7a,c). This was unexpected since for the FeNP-CUF system more flocs settled and were removed in the membrane tank because of the higher density of Fe_3_O_4_ nanoparticles in the flocs, and the fewer remaining flocs should have resulted in a reduced thickness of cake layer in FeNP-CUF system compared to that in the CUF system. The unexpected similarity in the cake thickness may be partly because the adhesion capacity of flocs onto the cake layer increased by the combined presence of Fe_3_O_4_ nanoparticles and more organic matter (such as EPS).

Comparing Fig. 7b with Fig. 7d, which show the cake layer cross-sections in greater resolution, there appeared to evidence of more EPS or other organic matter adsorbed from the raw water or produced by bacteria associated with the Fe_3_O_4_ nanoparticles in the FeNP-CUF cake layer. The organic matter is thought to come directly from the bacteria, which produced extracellular polysaccharide templates onto the Fe precipitates^43^,^44^,^45^. Also Fe_3_O_4_ nanoparticles may show a high bovine serum albumin (BSA) protein adsorption capacity in aqueous solution^6^, which caused a higher EPS concentration in the cake layer. It is suggested that a greater EPS concentration in the FeNP-CUF cake layer enhanced the adhesion ability of the cake layer, which was previously observed in wastewater treatment^46^, leading to fewer particles being removed during backwashing. This may be the reason that the thickness of the cake layer in the two systems was nearly the same, even though more flocs were settled in the FeNP-CUF membrane tank because of the higher density from the inclusion of Fe_3_O_4_ nanoparticles. The SEM images also suggested that some EPS existed on the membrane surface (Fig. 7b), which may aggravate the membrane fouling.

### Variation of TMP

As discussed previously, the different bacterial activity in the two systems resulted in a marked difference in membrane fouling. As the membrane flux of the FeNP-CUF and CUF streams were both set at a constant value, membrane fouling could be quantified by the temporal increase in TMP. The comparative increase in TMP for the FeNP-CUF and CUF streams is shown in Fig. 8, which covered an operating period of more than 30 days. It can be seen that the TMP increased with time from an initial value of approximately 5 kPa for both treatment streams.

It is clearly shown that the existence of Fe_3_O_4_ nanoparticles in the raw water produced an initial membrane fouling rate only a little greater than that without Fe_3_O_4_ (Fig. 8), but subsequently the TMP increased quickly from 15 days of operation to reach a TMP of 22 kPa after 25 days, which was much higher than that for the CUF system without nanoparticles (11.5 kPa). The importance of EPS in membrane bioreactor fouling was confirmed by Cho and Fane who determined that fouling occurred in two stages; a gradual deposition and sudden increase stage of biomass growth that required membrane cleaning^47^. This major difference was believed to be caused by biological effects which were discussed above with reference to various parameters that have revealed the membrane behaviour in detail. In particular, the presence of Fe_3_O_4_ nanoparticles most probably enhanced the bacterial activity.

After 25 days of operation, the membrane modules were taken out from the tanks and cleaned by high pressure tap water and sponge. On re-starting the treatment it was found that the initial TMP of the FeNP-CUF and CUF membranes was 8 kPa and 5.5 kPa, respectively. Since the CUF value was only slightly higher than that of a new membrane (5 kPa), we can conclude that the CUF membrane fouling during operation was mainly determined by the cake layer on the membrane surface, but for the FeNP-CUF membrane there was also some physical irreversible membrane fouling (~3 kPa). Therefore, the presence of Fe_3_O_4_ nanoparticles appears to cause both irreversible and reversible membrane fouling, and the association between Fe_3_O_4_ nanoparticles and enhanced bacterial activity, should be the main reason for this, based on the results from this investigation.

## Conclusions

In general, the benefits of applying coagulation as a pre-treatment for ultrafiltration are widely accepted. However, the presence of Fe_3_O_4_ nanoparticles in the raw water appears to be detrimental to UF performance by increasing the rate of membrane fouling. The reasons for this behavior and specific conclusions of this study are summarized as follows:

1. While the presence of Fe_3_O_4_ nanoparticles had no adverse impact on the coagulation performance, their presence within residual coagulant flocs led to their accumulation in the cake layer on the surface of membrane and an increase in fouling effects. It was observed that the nature of the accumulated Fe-solids changed with time, and was linked to increased bacterial activity.
2. The rate of membrane fouling (TMP increase) was similar in the early stages of the UF runs for the two raw waters, but it increased rapidly after about 15 days for the raw water containing Fe_3_O_4_ nanoparticles, suggesting the involvement of biological effects. An increase in microbial activity can enhance both reversible fouling (changing the properties of the cake layer) and irreversible fouling (blockage of membrane pores).
3. For the FeNP-CUF system, the sizes of precipitate nanoparticles were smaller and the cake layer appeared to have a relatively lower porosity, compared to the CUF system. The internal fouling induced by pore deposition or blockage of the FeNP-CUF membrane was much more serious than the CUF membrane, as indicated visually by SEM and by the greater rate of TMP increase.
4. The presence of increased microbial activity in the FeNP-CUF system was evident from the measured concentrations of EPS and DNA, and fluorescence intensities. It is speculated that microorganisms catalyze Fe_3_O_4_ nanoparticles to iron oxyhydroxides, thereby leading to increased bacterial growth. Associated with the bacterial growth is the production of EPS which enhances the bonding with, and between, the coagulant flocs, giving rise to a lower porosity, more resilient cake layer.

## Additional Information

**How to cite this article**: Yu, W. *et al.* Contribution of Fe_3_O_4_ nanoparticles to the fouling of ultrafiltration with coagulation pre-treatment. *Sci. Rep.* **5**, 13067; doi: 10.1038/srep13067 (2015).

## Supplementary Material

Supplementary (Figure S1)

## Figures and Tables

**Table 1:** Quality parameters for raw water and UF influents^a^/filtrates.

Parameter^b^	Raw water		Membrane tank		UF filtrate	
	CUF	FeNP-CUF	CUF	FeNP-CUF	CUF	FeNP-CUF
UV_254_ (cm^−1^)	0.098 ± 0.006	0.098 ± 0.006	0.028 ± 0.002	0.032 ± 0.002	0.028 ± 0.003	0.031 ± 0.002
TOC (mg/L)	3.80 ± 0.42	3.85 ± 0.42	2.38 ± 0.18	2.52 ± 0.224	2.17 ± 0.23	2.02 ± 0.15
Turbidity (NTU)	3.28 ± 0.16	6.43 ± 0.16	2.67 ± 0.28	2.36 ± 0.35	0.02 ± 0.02	0.03 ± 0.03
P (mg/L)	0.322 ± 0.035	0.374 ± 0.047	0.078 ± 0.002	0.083 ± 0.008	0.065 ± 0.002	0.070 ± 0.005
NO_3_^−^-N (mg/L)	2.35 ± 0.58	2.56 ± 0.67	2.70 ± 0.49	2.78 ± 0.55	2.71 ± 0.27	2.81 ± 0.45
NH_4_^+^-N (mg/L)	0.37 ± 0.05	0.32 ± 0.05	nd^c^	nd	nd	nd
Fe (mg/L)	0.071 ± 0.014	2.023 ± 0.243	0.063 ± 0.011	0.131 ± 0.034	0.023 ± 0.005	0.041 ± 0.008
pH	7.85 ± 0.06	7.87 ± 0.06	7.33 ± 0.04	7.34 ± 0.04	7.38 ± 0.05	7.40 ± 0.03

^a^Influent—within membrane tank, immediately after flocculation units.

^b^For turbidity, UV_254_, and DOC, the number of measurements, n = 9; for residual P, NO_3_^−^-N and NH_4_^+^-N, n = 5.

^c^nd—concentration below detection limit.
