# Supplementary material for: Contribution of Fe3O4 nanoparticles to the fouling of ultrafiltration with coagulation pre-treatment
Source: Sci Rep. 2015 Aug 13;5:13067. doi: 10.1038/srep13067 (PMC4535038; doi:10.1038/srep13067)
Supplement: Supplementary (Figure S1) [file srep13067-s1.doc]

**Supplementary**

**Contribution of Fe_3_O_4_ nanoparticles to the fouling of ultrafiltration with coagulation pre-treatment**

*Wenzheng Yu^a,b#^*, *Lei Xu^a,c#^*, *Nigel Graham^b^*, *Jiuhui Qu^a*^*

*^a^ Key Laboratory of Aquatic Chemistry, Research Center for Eco-Environmental Sciences, Chinese Academy of Sciences, Beijing 100085, China*

*w.yu@imperial.ac.uk, jhqu@rcees.ac.cn*

*^b^ Department of Civil and Environmental Engineering, Imperial College London, South Kensington Campus, London SW7 2AZ, UK.*

[*n.graham@imperial.ac.uk*](mailto:n.graham@imperial.ac.uk)

*^c^ Centre for Water Resources Research (CWRR), School of Civil, Structural and Environmental Engineering, University College Dublin, Newstead Building, Belfield, Dublin 4, Ireland*

*leixu.rcees@gmail.com*

*^#^* *These authors contributed equally to this work*

**Corresponding author: Tel: +86 10 62849128; Fax: +86 10 62849160*

**Figure S1** XRD pattern of Fe_3_O_4_ nanoparticles
